# Genetic and Transcriptomic Analyses of the Purple Coloration Trait in the Inbred Line 7A01 of *Brassica rapa*

**DOI:** 10.3390/plants15142192

**Published:** 2026-07-17

**Authors:** Bin Zhang, Xiaohui Cui, Shaojie Ma, Ziheng Zhang, Zijin Liu, Mingxun Chen, Yuan Guo, Saiqi Yang

**Affiliations:** State Key Laboratory for Crop Stress Resistance and High-Efficiency Production, National Yangling Agricultural Biotechnology & Breeding Center, Shaanxi Key Laboratory of Crop Heterosis, College of Agronomy, Northwest A&F University, Yangling 712100, China; q16890203@163.com (B.Z.); 18248047704@163.com (X.C.); 18639154863@163.com (S.M.); ziheng0306@nwafu.edu.cn (Z.Z.); liuzijin@nwafu.edu.cn (Z.L.); cmx786@nwafu.edu.cn (M.C.)

**Keywords:** *Brassica rapa*, anthocyanin, gene mapping, molecular marker development, transcriptome

## Abstract

Anthocyanins are highly valued water-soluble pigments that play pleiotropic roles in plant growth and development, contributing to various physiological and ecological functions. Although multiple *Brassica rapa* varieties have been reported to accumulate anthocyanins in different tissues, the genes controlling this trait remain largely elusive. In this study, we characterized the *B. rapa* inbred line 7A01, which exhibits purple coloration in leaf veins, petioles, and flowering stalks, a trait later confirmed to be associated with high anthocyanin content. Genetic analysis indicates that this trait behaves as a monogenic trait, possibly controlled by a single incompletely dominant nuclear locus, which was mapped to a 5.1 cM region on chromosome A10 using a *Brassica* 50K SNP array combined with bulked segregant analysis. Five simple sequence repeat markers linked to the purple coloration trait were developed. Transcriptome profiling of shoot apices and flowering stalks identified 3953 and 5922 differentially expressed genes (DEGs) involved in anthocyanin biosynthesis, respectively. These DEGs included both structural genes and transcription factors, and their expression was validated by RT-qPCR. Our findings provide a genetic and molecular framework for the purple coloration trait in *B. rapa* and offer candidate genes for future breeding efforts.

## 1. Introduction

Anthocyanins are water-soluble pigments that belong to the flavonoid class of secondary metabolites, contributing red, purple, and blue pigmentation that attracts pollinators and seed dispersers in various plant organs including flowers, leaves, seeds, and fruits [1,2]. Anthocyanins are also widely used as natural food colorants [1,3]. The biosynthesis of anthocyanins is influenced by environmental cues, such as ultraviolet radiation [4], temperature [5,6], cold, salt, and drought stresses [7], nutrient deficiency [8], and pathogens [9], and anthocyanin accumulation is increasingly recognized as part of broader stress-responsive metabolic reprogramming and defense adaptation networks in plants [6,10,11,12]. Moreover, anthocyanins have been reported to be beneficial in the prevention of oxidative stress-related cardiovascular and neurodegenerative diseases [3,13]. Therefore, it is of both scientific and economic importance to elucidate the mechanisms that modulate anthocyanin biosynthesis.

Anthocyanins are flavonoid derivatives produced through the phenylpropanoid pathway and require sequential action of a list of structural enzymes, including phenylalanine ammonium lyase (PAL), cinnamate 4-hydroxylase (C4H), 4-coumarate: CoA ligase (4CL), naringenin-chalcone synthase (CHS), chalcone isomerase (CHI), flavanone 3-hydroxylase (F3H), dihydroflavonol 4-reductase (DFR), anthocyanidin synthase (ANS), anthocyanin-related glutathione S-transferase (arGST), and UDP-dependent flavonoid-3-O-glycosyltransferase (UFGT) [1,2,14]. Flavonoid 3′-hydroxylase (F3′H) and flavonoid 3′,5′-hydroxylase (F3′5′H) are responsible for adding additional hydroxyl groups on the B-ring to generate different anthocyanin species, including pelargonidin, cyanidin, and delphinidin derivatives [15], that determine the color ranging from orange-red to blue [2,3].

The expression of these anthocyanin biosynthesis genes is subjected to sophisticated spatiotemporal regulation. This includes members of MYB transcription factors (TFs) such as TRANSPARENT TESTA2 (TT2) and PRODUCTION OF ANTHOCYANIN PIGMENT1 (PAP1/MYB75), basic helix-loop-helix (bHLH) TFs such as TT8 and GLABRA3, as well as WD40 TFs such as TRANSPARENT TESTA GLABRA1 (TTG1), which form an MBW protein complex [2,16,17,18,19]. The regulation of MBW complex in anthocyanin biosynthesis was reported to be conserved in pigment model plants such as *Zea mays*, *Antirrhinum majus*, and *Petunia hybrida* [17,20,21]. Moreover, the expression of WRKY TF *TTG2* is directly activated by MBW complex TT2-TT8-TTG1, and TTG2 physically interacts with TTG1 to enhance their upregulation of anthocyanin biosynthesis genes, including vacuolar transport-associated MATE-type transporter *TT12* and P-type ATPase proton pump *TT13* [22,23]. In contrast to the highly conserved activation complex, a wide variety of anthocyanin biosynthesis repressors, including MYB, SQUAMOSA PROMOTER BINDING PROTEIN-LIKE, HOMEODOMAIN-LEUCINE ZIPPER, NAC, and TCP TFs, Skp1/Cullin/F-box (SCF) E3 ubiquitin ligase complex, as well as small RNA families, were also reported to mediate anthocyanin accumulation in different tissues and in response to various developmental and environmental cues (reviewed in LaFountain and Yuan, 2021 [24]).

*Brassica rapa* has formed diverse morphological types during domestication and environmental selection, including oil-type rapeseed, root-type, and leaf-type vegetables [25]. Among these, various varieties with anthocyanin accumulation in different tissues were reported, including but not limited to purple leaf vein, petiole, and stalk (*B. campestris* L. *var. purpurea Bailey*) [26], purple edible leaves in bok choy (*B. rapa var chinensis*) [27], swollen roots in ‘Tsuda’ turnip (*B. rapa* subsp. *rapa*) [28], purple sprout in Pak Choi (*B. rapa* L. ssp. *chinensis*) [29], hypocotyl-tuber in turnip (*B. rapa* ssp. *rapa*) [30,31], midribs and leaves in seedlings of purple head Chinese cabbage [32,33], non-heading Chinese cabbage [34], and purple cabbage ‘Zicaitai’ (*B. rapa var. purpuraria*) [35].

Genome-wide studies have been conducted to identify anthocyanin biosynthesis genes in *B. rapa*. Using homologous genes in *A. thaliana* as queries, a total of 73 anthocyanin biosynthesis genes in *B. rapa* were identified, representing 39 out of the 41 homologs in *A. thaliana*, and most genes exist in more than one copy [36]. By RNA sequencing (RNA-seq) and Weighted Gene Co-expression Network Analysis (WGCNA) using sprouts of purple pak choi (*B. rapa* L. ssp. *chinensis*), a recent study unveiled that *BraPAP2.A03*, *BraTT8.A09*, *BraMYBL2.A07*, and their potential target *BraDFR.A09* are key hub genes in anthocyanin biosynthesis [29]. Recently, more hub genes were identified to be responsible for anthocyanin biosynthesis in *B. rapa*, e.g., homologs of *CHI* (*BraA03g059660.3C*) in non-heading Chinese cabbage [34], *arGST* (*BraA10g022830.3.5C*) in ‘Zicaitai’ (*B. rapa var. purpuraria*)) [37], *MYB32* (Genbank XM_009109115) in Chinese cabbage [38], *bHLH* (*BraA05g014086E*), and *WRKY* (*BraA05g014093E*) in turnip (*B. rapa* ssp. *rapa*) [30]. However, our understanding of key genes determining anthocyanin accumulation in *B. rapa*, particularly in different tissues and at various developmental stages, remains far from complete.

In this study, we focused on an inbred line, 7A01, which shows purple leaf veins, petioles, and flowering stalks. To identify the genetic basis of this trait, we generated a recombinant inbred line (RIL) population derived from a cross between 7A01 and the green cultivar ‘Jinqiu 66’. Genetic analysis indicated that this trait behaves as a single incompletely dominant nuclear locus. By applying bulked segregant analysis (BSA), this locus was mapped to a 5.1 cM region on chromosome A10, and five simple sequence repeat (SSR) markers were developed. Moreover, by performing transcriptome analyses with the shoot apices and flowering stalks of 7A01 and ‘Jinqiu 66’, multiple differentially expressed genes (DEGs) related to anthocyanin biosynthesis were identified. Our study provides new insight into the genetic and molecular basis of the purple leaf vein, petiole, and flowering stalk trait in *B. rapa*.

## 2. Results

### 2.1. The Purple Coloration Trait in B. rapa Inbred Line 7A01 Is Associated with Increased Anthocyanin Accumulation

**Figure 1:**
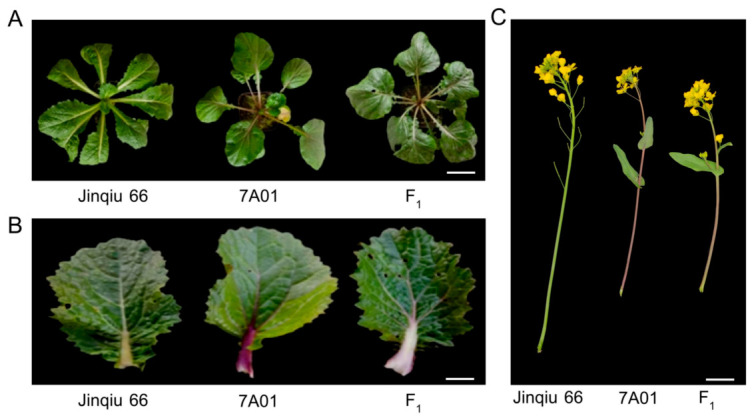
Color characteristics of Jinqiu 66, 7A01, and their F_1_ hybrid offspring during the seedling stage and flowering stage. (**A**) Representative images of indicated lines at the five-leaf stage. Scale bars = 2 cm. (**B**) Color characteristics of petioles and leaf veins from the fifth true leaf of the indicated lines. Scale bars = 1 cm. (**C**) Color characteristics of stems at the flowering stage of the indicated lines. Scale bars = 5 cm.

In this study, the *B. rapa* purple flowering stalk inbred line 7A01 and the green cultivar ‘Jinqiu 66’ were used as parental materials to explore the potential candidate genes associated with the purple flowering stalk trait (Figure S1). A closer phenotypic analysis indicated that 7A01 showed purple coloration in petioles and leaf veins at the five-leaf stage (Figure 1A,B) and in the flowering stalk at the bolting stage (Figure 1C and Figure S1). 7A01 and ‘Jinqiu 66’ were crossed, and the offspring, i.e., the generated F_1_ population, also showed similar purple coloration in the petioles, leaf veins, and flowering stalks, with the color being lighter than that of the parental line 7A01 (Figure 1 and Figure S1).

**Figure 2:**
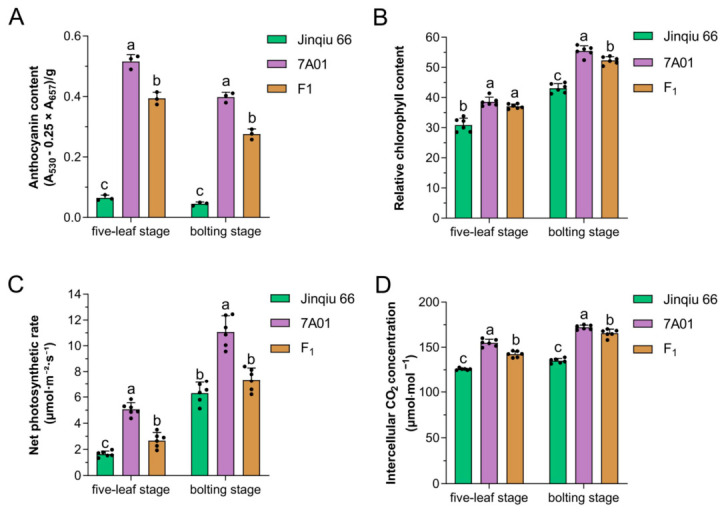
Physiological characteristics of Jinqiu 66, 7A01, and their F_1_ hybrid offspring. Anthocyanin contents (**A**), relative chlorophyll contents (**B**), net photosynthetic rate (**C**), and intercellular CO_2_ concentration (**D**) of the indicated lines at the five-leaf stage and bolting stage. Values are means ± SD. Different letters indicate significant differences (one-way ANOVA with Tukey’s test, *p* < 0.05).

We then analyzed whether the purple coloration in 7A01 was related to anthocyanin accumulation. Quantitative analyses revealed that anthocyanin contents in the petioles at the five-leaf stage and the flowering stalks at the bolting stage were substantially higher in 7A01 than in ‘Jinqiu 66’, which showed only basal levels of anthocyanin contents (Figure 2A). This pattern aligns with the purple and green phenotypes of 7A01 and ‘Jinqiu 66’, respectively (Figure 1 and Figure S1). Consistently, their F_1_ hybrid exhibited intermediate anthocyanin levels at both stages (Figure 2A). These results suggested that the purple coloration in 7A01 was likely caused by the accumulation of anthocyanins. Additionally, the relative chlorophyll contents of 7A01 at both stages were moderately but significantly higher than ‘Jinqiu 66’ and the F_1_ hybrid, while ‘Jinqiu 66’ showed the lowest relative chlorophyll content (Figure 2B).

Previous studies indicated that light-induced anthocyanins play a protective role in photosynthetically active plant organs against excessive light intensities and oxidative stress [2,39,40,41,42]. We further explored the differences in photosynthetic efficiency between 7A01 and Jinqiu 66. The results showed that net photosynthetic rate (*Pn*) and intercellular CO_2_ concentration (*Ci*) in the petioles at the five-leaf stage and the flowering stalks at the bolting stage were significantly higher in 7A01 than those of ‘Jinqiu 66’, while the F_1_ hybrid exhibited intermediate levels (Figure 2C,D). These results suggest that, besides the accumulation of anthocyanins, 7A01 also showed higher photosynthetic efficiency than Jinqiu 66. Further studies are needed to rule out the influences of genetic background differences between these two lines.

### 2.2. Genetic Inheritance of the Purple Coloration Trait of B. rapa Inbred Line 7A01

To investigate the genetic inheritance of the purple coloration trait, using 7A01 (purple) and ‘Jinqiu 66’ (green) as parental lines, F_1_, F_2_, and BC_1_ segregating populations were constructed. During the two consecutive years of field phenotypic observations in 2024 and 2025, it was found that the purple, light purple, and green coloration traits on the leaf veins, petioles, and flowering stalks consistently appeared simultaneously in the obtained populations. Subsequently, we analyzed the phenotypic segregation of the purple, light purple, and green coloration traits in the F_2_ and BC_1_ populations (Table 1). The chi-square tests indicated that the ratio of green plants to purple (including light purple) plants in the F_2_ population followed a 1:3 segregation ratio, while the ratio in the BC_1_ population followed a 1:1 segregation ratio. In conclusion, these results suggest that the purple coloration trait of 7A01 is a single Mendelian genetic trait, possibly controlled by a single nuclear locus, without cytoplasmic effects, and this purple coloration trait is incompletely dominant (Table 1).

### 2.3. Mapping of the Genomic Locus Linked to the Purple Coloration Trait

We then proceeded to localize the genomic locus linked to the purple coloration trait of 7A01. A high-density *Brassica* 50K Illumina Infinium™ SNP array was employed, in combination with the bulked segregant analysis (BSA) and collinearity analysis, to screen for SNP markers and preliminarily map the purple coloration trait in 7A01. SNP array analysis identified four polymorphic SNP loci between 7A01 and ‘Jinqiu 66’ from genome-wide distributed SNP markers, among which one is located on the A01 chromosome of *B. napus*, and three are located on the A10 chromosome of *B. napus* (Supplementary Table S1). Through collinearity comparison, the 4.43–4.46 Mb region on the A10 chromosome of *B. napus* was mapped to the 28.15–29.26 Mb region on the A10 chromosome of *B. rapa* (Supplementary Tables S1 and S2).

**Figure 3:**
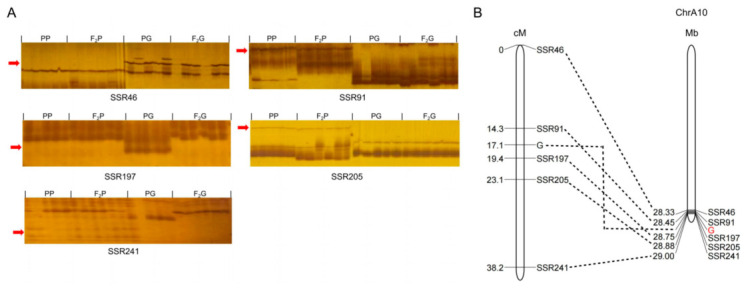
Localization of the genomic region that co-segregates with the purple coloration trait. (**A**) Verification results of SSR markers in parental plants and F_2_ plants with green stalk and purple stalk traits. PP: DNA from 5 individual parental plants with purple coloration; PG: DNA from 5 individual parental plants with green coloration; F_2_P: DNA from 6 individual plants with purple coloration in the F_2_ generation; F_2_G: DNA from 6 individual plants with green coloration in the F_2_ generation. Arrows indicate the target bands. (**B**) Genetic map and physical map of candidate genes related to the purple coloration trait.

Within the target interval, 109 pairs of SSR primers aiming for the 28.15–29.26 Mb region on the A10 chromosome of *B. rapa* genome were designed. Meanwhile, 3 pairs of intron polymorphism (IP) primers were designed aiming for the 5.21–5.23 Mb region on the A01 chromosome of *B. rapa* genome. Among these, five SSR primers (SSR46, SSR91, SSR197, SSR205, and SSR241) exhibited stable polymorphism in a small population (Figure 3A), while 3 pairs of IP primers showed no polymorphism. These five stable SSR markers were used to genotype 611 individuals from the F_2_ population, and the genetic distances between the markers and the target genomic region were calculated based on the number of recombinant individuals detected. Ultimately, the target genomic region was confined to a 5.1 cM interval on chromosome A10 of *B. rapa* genome (Figure 3B). The closest flanking markers, SSR91 and SSR197, were located at physical distances of 28,453,405 bp and 28,749,597 bp, respectively, with a physical distance of 296 kb between them (Figure 3B). Within this region, a total of 46 genes were identified based on the functional descriptions of their *A. thaliana* homologs in TAIR (https://www.arabidopsis.org/) (Supplementary Table S3). Notably, none of the identified genes are previously reported to be involved in anthocyanin biosynthesis.

### 2.4. Identification of Candidate Genes Associated with Anthocyanin Accumulation in Petioles and Leaf Veins from B. rapa Inbred Line 7A01

**Figure 4:**
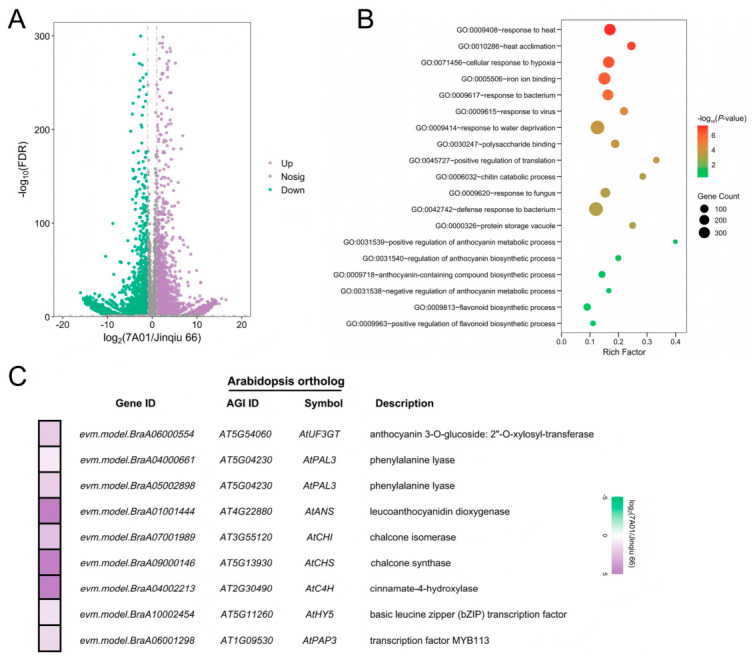
Transcriptome analysis of shoot apices from Jinqiu 66 and 7A01 at the five-leaf stage. (**A**) Volcano plot showing total gene profiles comparing Jinqiu 66 and 7A01. Purple points represent upregulated genes, green points represent downregulated genes, and grey points represent non-differentially expressed genes. (**B**) GO functional enrichment analysis of DEGs in shoot apices from Jinqiu 66 and 7A01 at the five-leaf stage. The rich factor for the *X*-axis represents the ratio of DEGs to the total number of annotated genes in a given pathway; the *Y*-axis represents the name of the enriched pathway; the color scale indicates different thresholds of the *p*-value; the size of the dots represents the number of genes corresponding to each pathway. (**C**) Heatmap of DEGs related to anthocyanin biosynthesis in shoot apices of 7A01 at the five-leaf stage based on transcriptome analysis. The purple-to-green gradient bars indicate |log_2_(7A01/Jinqiu 66)| of each gene. FDR, false discovery rate; DEGs, differentially expressed genes; GO, gene ontology.

To identify the candidate genes associated with the purple coloration in petioles and leaf veins of 7A01, a transcriptome analysis was performed first with shoot apices from 7A01 and ‘Jinqiu 66’ at the five-leaf stage. A total of 3953 DEGs were identified when comparing 7A01 with ‘Jinqiu 66’, among which 2393 DEGs were upregulated, and 1560 DEGs were downregulated (Figure 4A). Gene ontology (GO) analysis was conducted to elucidate the biological processes involved in this process (Figure 4B). The result revealed significant enrichment of multiple anthocyanin accumulation-related GO entries, including positive regulation of anthocyanin metabolic process (GO:0031539), regulation of anthocyanin biosynthetic process (GO:0031540), and anthocyanin-containing compound biosynthetic process (GO:0009718) (Figure 4B). Interestingly, negative regulation of anthocyanin metabolic process (GO:0031538) was also enriched. Furthermore, GO entries related to flavonoid biosynthesis, such as flavonoid biosynthetic process (GO:0009813) and positive regulation of flavonoid biosynthetic process (GO:0009963), were also significantly enriched (Figure 4B). Additionally, GO entries related to abiotic stress responses, such as response to heat (GO:0009408), heat acclimation (GO:0010286), cellular response to hypoxia (GO:0071456), and response to water deprivation (GO:0009414), as well as GO entries related to biotic stress responses, including response to bacterium (GO:0009617), defense response to bacterium (GO:0042742), response to virus (GO:0009615), and response to fungus (GO:0009620), were also significantly enriched (Figure 4B).

**Figure 5:**
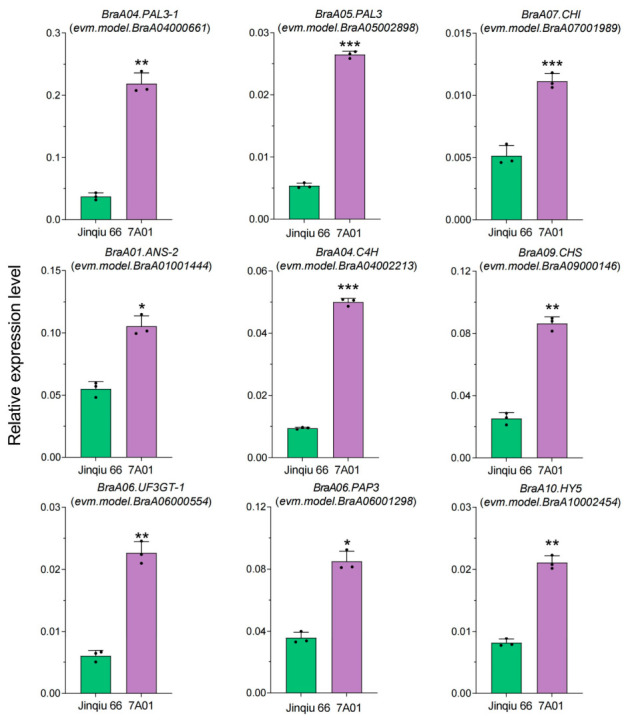
Verification of DEGs related to anthocyanin accumulation in shoot apices of 7A01 at the five-leaf stage. RT-qPCR validation of selected differentially expressed genes in shoot apices of Jinqiu 66 and 7A01 at the five-leaf stage. Results were normalized to *BraGAPDH* as an internal control. Values are means ± SD (*n* = 3). Asterisks indicate significant differences compared with Jinqiu 66 (two-tailed Student’s *t*-test, * *p* < 0.05, ** *p* < 0.01, *** *p* < 0.001). DEGs, differentially expressed genes.

Based on the results of GO analysis, combined with the previously reported functions of homologous genes in *A. thaliana*, genes related to the anthocyanin biosynthesis pathway were identified (Figure 4C), including structural genes homologous to *AtUF3GT*, *AtPAL3*, *AtANS*, *AtCHI*, *AtCHS*, and *AtC4H*, as well as TFs homologous to *ELONGATED HYPOCOTYL 5* (*AtHY5*) and *AtPAP3*, which positively regulate anthocyanin biosynthesis (Figure 4C). Real-time quantitative PCR (RT-qPCR) analyses were then performed to verify the upregulation of these genes. The results showed that all nine tested *B. rapa* genes were significantly upregulated in the shoot apices of 7A01 (Figure 5). These results imply that upregulation of these genes might be associated with the accumulation of anthocyanin in petioles and leaf veins of 7A01.

### 2.5. Identification of Candidate Genes Associated with Anthocyanin Accumulation in Flowering Stalks from B. rapa Inbred Line 7A01

**Figure 6:**
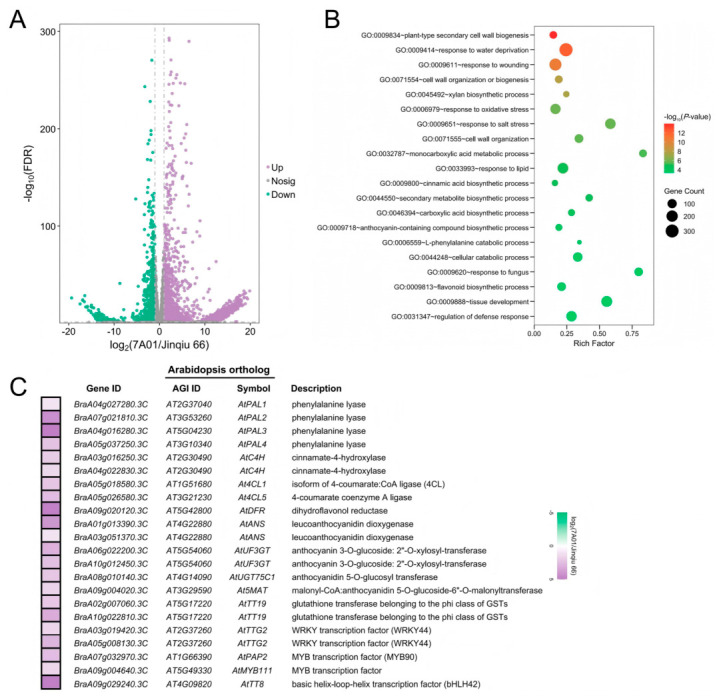
Transcriptome analysis of flowering stalks from Jinqiu 66 and 7A01 at the bolting stage. (**A**) Volcano plot showing total gene profiles comparing Jinqiu 66 and 7A01. Purple points represent upregulated genes, green points represent downregulated genes, and grey points represent non-differentially expressed genes. (**B**) GO functional enrichment analysis of DEGs in flowering stalks from Jinqiu 66 and 7A01. The rich factor for the *X*-axis represents the ratio of DEGs to the total number of annotated genes in a given pathway; the *Y*-axis represents the name of the enriched pathway; the color scale indicates different thresholds of the *p*-value; the size of the dots represents the number of genes corresponding to each pathway. (**C**) Heatmap of DEGs related to anthocyanin biosynthesis in flowering stalks of 7A01 based on transcriptome analysis. The purple-to-green gradient bars indicate |log_2_(7A01/Jinqiu 66)| of each gene. FDR, false discovery rate; DEGs, differentially expressed genes; GO, gene ontology.

**Figure 7:**
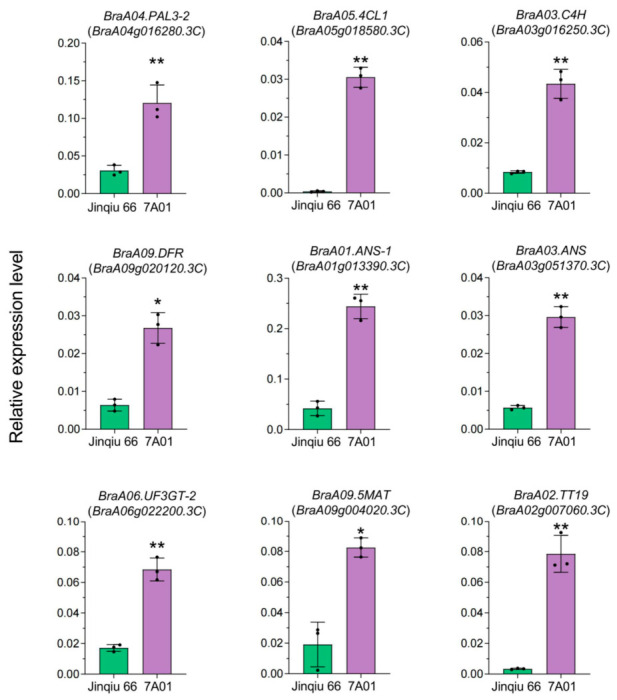
Verification of DEGs related to anthocyanin accumulation in flowering stalks of 7A01 at the bolting stage. RT-qPCR validation of selected differentially expressed genes in flowering stalks of Jinqiu 66 and 7A01. Results were normalized to *BraGAPDH* as an internal control. Values are means ± SD (*n* = 3). Asterisks indicate significant differences compared with Jinqiu 66 (two-tailed Student’s *t*-test, * *p* < 0.05, ** *p* < 0.01). DEGs, differentially expressed genes.

Next, a transcriptome analysis was performed with flowering stalks from 7A01 and ‘Jinqiu 66’ to identify the candidate genes associated with the purple coloration in flowering stalks of 7A01. A total of 5922 DEGs were identified when comparing 7A01 with ‘Jinqiu 66’, among which 2949 DEGs were upregulated, and 2973 DEGs were downregulated (Figure 6A). Resembling the results with shoot apices (Figure 4B), GO analysis revealed significant enrichment of anthocyanin-containing compound biosynthetic process (GO:0009718) and GO entries related to biosynthesis of flavonoid and other anthocyanin precursors, including cinnamic acid biosynthetic process (GO:0009800), L-phenylalanine catabolic process (GO:0006559), and flavonoid biosynthetic process (GO:0009813) (Figure 6B). Furthermore, GO entries related to abiotic and biotic stress responses, such as response to water deprivation (GO:0009414), response to wounding (GO:0009611), response to oxidative stress (GO:0006979), response to salt stress (GO:0009651), regulation of defense response (GO:0031347), and response to fungus (GO:0009620), were also significantly enriched (Figure 6B). Screening of genes related to the anthocyanin biosynthesis pathway identified structural genes homologous to *AtPAL1*, *AtPAL2*, *AtPAL3*, *AtPAL4*, *AtC4H*, *At4CL1*, *At4CL5*, *AtDFR*, *AtANS*, *AtUF3GT*, *AtUGT75C1*, *At5MAT*, and *AtTT19*, as well as TFs homologous to *AtTTG2*, *AtPAP2*, *AtMYB111*, and *AtTT8* (Figure 6C). Using flowering stalks of 7A01 and ‘Jinqiu 66’, we then selected nine DEGs for verification with RT-qPCR (Figure 7), including four *B. rapa* genes that are homologous to *AtPAL3*, *AtC4H*, *AtANS*, and *AtUF3GT*, which were previously verified in petioles and leaf veins (Figure 5). The results showed that, consistent with the RNA-seq result (Figure 6), all selected genes were significantly upregulated in 7A01 (Figure 7). Similarly, our results imply that the promoted expression of these genes might be associated with the purple flowering stalk trait in *B. rapa* inbred line 7A01.

## 3. Discussion

The biosynthesis of anthocyanins is widely studied in many organisms due to its pleiotropic role in plant growth and development, as well as its nutritional, medical, and economic value [1,2,3]. Although plenty of studies regarding the biosynthesis and applications of anthocyanins in *Brassica* crops have been reported [13,30,34,37,38,43], the complete picture of anthocyanin biosynthesis in *B. rapa* is still far from fully understood. In this study, we identified that anthocyanin accumulation in the leaf veins, petioles, and flowering stalks of *B. rapa* inbred line 7A01 is possibly controlled by a single incompletely dominant nuclear locus, which was mapped to a 5.1 cM region on chromosome A10 (Figure 1, Figure 2 and Figure 3). Moreover, multiple anthocyanin biosynthesis-related genes that might contribute to the purple coloration trait in 7A01 were identified by transcriptomic analyses (Figure 4, Figure 5, Figure 6 and Figure 7). In addition, we found that 7A01 also exhibits higher relative chlorophyll content and photosynthetic efficiency than Jinqiu 66, with the F_1_ hybrid exhibiting intermediate levels (Figure 2B–D). Further analysis is required to determine the relationship between anthocyanin accumulation and these physiological characteristics in these lines in future studies.

Previous studies reported the endeavors of localizing the causal loci for anthocyanin accumulation in *B. rapa*, and varying results were obtained. For example, the *anl* locus responsible for the anthocyaninless trait in stems of rapid-cycling *B. rapa* is associated with linkage group R9 of *B. rapa* reference map [44]. Another study showed that the red-skinned swollen root and petiole trait in a doubled haploid turnip line (*B. rapa* cv. ‘Iyo-hikabu’) associates with a monogenic locus *Anp* in linkage group R07 of the *B. rapa* [45]. A later study indicated that the purple leaf color trait of Chinese cabbage is associated with a single dominant gene, *BrPur*, on linkage group A03 of *B. rapa* [46]. The application of whole genome sequencing helped fine-mapping of the causal genes, e.g., sequencing of two bulked pools of anthocyanin-defective turnip (*Brassica rapa* subsp. *rapa*) mutant *w68* mapped R2R3 MYB TF *BrPAP1a* on chromosome A07 to be responsible for the purple peel of swollen root trait [28]. A recent study with BSA-sequencing of recombinant inbred line (RIL) population fine-mapped *BraP2*, a locus on chromosome A03 associated with purple leaf coloration of non-heading Chinese cabbage (*B. rapa*), and the causal gene *BraCHI* (*BraA03g059660.3C*) locates within the 65.31 kb candidate region [34]. In this study, we showed that the anthocyanin accumulation trait in leaf veins, petioles, and flowering stalks of the *B. rapa* inbred line 7A01 is controlled by a single incompletely dominant nuclear locus (Figure 1 and Figure 2A, Table 1). Using a *Brassica* 50K SNP array combined with BSA, the locus was mapped to a 5.1 cM region on chromosome A10 (Figure 3, Supplementary Tables S1 and S2). Five linked SSR markers were developed, and the closest flanking markers defined a physical interval of 296 kb containing 46 genes annotated based on their *A. thaliana* homologs in TAIR (Figure 3, Supplementary Table S3). Notably, none of these are homologous to known anthocyanin biosynthesis genes. Although a high number of predicted anthocyanin biosynthesis-related genes were reported in the previous genome-wide studies in *B. rapa* [29,36], the overall regulatory mechanisms underlying anthocyanin accumulation in this species remain far from complete. It is therefore likely that additional loci/genes contributing to this process in *B. rapa* remain to be discovered. Further studies would be needed to pin down the potential causal gene within the identified interval.

The similar GO enrichment patterns between comparative transcriptomes of shoot apices at the five-leaf stage and flowering stalks at the bolting stage suggest that the purple coloration in these two stages involves comparable biological processes, including GO entries related to anthocyanin biosynthesis and flavonoid biosynthesis (Figure 4A,B and Figure 6A,B), consistent with the previous consensus that anthocyanin biosynthesis is a branch in the flavonoid biosynthesis [1,2,13,47]. Moreover, both GO analyses revealed enrichment of biological processes related to biotic and abiotic stress responses (Figure 4A,B and Figure 6A,B), aligning with the previously reported correlation between anthocyanin accumulation and stress responses in previous reports [1,2,5,6,7,9,10,11,13,47,48]. Further analysis is needed to verify whether increased anthocyanin accumulation in 7A01 affects its stress responses in future studies.

Furthermore, transcriptome results of shoot apices and flowering stalks identified multiple anthocyanin biosynthesis-related genes, among which nine *B. rapa* genes that are homologous to *AtPAL3* (*evm.model.BraA04000661*, *evm.model.BraA05002898*, and *BraA04g016280.3C*), *AtC4H* (*evm.model.BraA04002213* and *BraA03g016250.3C*), *AtANS* (*evm.model.BraA01001444* and *BraA01g013390.3C*), and *AtUF3GT* (*evm.model.BraA06000554* and *BraA06g022200.3C*) were upregulated in both transcriptome and RT-qPCR analyses (Figure 4C, Figure 5, Figure 6C and Figure 7), suggesting that anthocyanin accumulation in shoot apices and flowering stalks in 7A01 might be achieved via upregulation of a set of similar but not identical anthocyanin biosynthesis structural genes.

On the other hand, *BraA10.HY5* (*evm.model.BraA10002454*) and *BraA06.PAP3* (*evm.model.BraA06001298*) were upregulated exclusively in shoot apices of 7A01 (Figure 4 and Figure 5). These two genes are homologous to previously reported *AtHY5* [5,43,48,49] and *AtPAP3*/*MYB113* [50,51], which were shown to promote anthocyanin biosynthesis. In comparison, in addition to more upregulated anthocyanin biosynthetic genes exclusively upregulated in flowering stalks (e.g., genes homologous to *AtPAL1*, *AtPAL2*, *AtPAL4*, *At4CL1*, *At4CL5*, and *AtDFR*), genes involved in anthocyanin decoration and transport, including homologs of *AtUGT75C1* (encoding anthocyanidin 5-O-glucosyl transferase [16,52]), *At5MAT* (encoding anthocyanin 5-glucoside malonyltransferase [53]), and *AtTT19* (encoding glutathione S-transferase that functions in both anthocyanin biosynthesis [14] and transport [54]), were also upregulated only in flowering stalks (Figure 4C, Figure 5, Figure 6C and Figure 7). Moreover, genes homologous to TFs that were previously reported to promote anthocyanin biosynthesis, such as *AtTTG2* [22,23], *AtPAP2* [50,51], *AtMYB111* [50,55], and *AtTT8* [18,22,23,28], were exclusively upregulated in flowering stalks (Figure 4C and Figure 6C).

The differences in upregulated anthocyanin biosynthetic genes, particularly in TFs (Figure 4C and Figure 6C), align with the previous reports that transcriptional control of pigmentation might show tissue-specific patterns. For instance, AtTT8, AtTT2, and AtTTG1 were shown to specifically control proanthocyanidin accumulation in the seed coat [18]. Similarly, the *ttg2* mutant pigment phenotype is restricted to the seed coat, with no loss of anthocyanins in the plant body [17,23]. In *Petunia*, the PH4 (MYB)–AN1 (ANTHOCYANIN1, bHLH)–AN11 (WD40) complex and its direct downstream target PH3 (a homolog of AtTTG2) function specifically in the control of flower color [20]. Since our mapping results revealed no known anthocyanin biosynthesis-related gene within the confined 296 kb region (Figure 3, Supplementary Table S3), it is possible that the purple coloration in 7A01 is potentially associated with a new candidate gene that specifically controls anthocyanin accumulation in leaf veins, petioles, and flowering stalks. For example, three among the 46 genes encode TFs (Supplementary Table S3): *BraA10g029490* (Nuclear Factor Y TF), *BraA10g030030* (zinc finger TF), and *BraA10g029770* (PHR1-LIKE TF), which might play a regulatory role associated with the purple coloration trait in 7A01. On the other hand, other mechanisms cannot be excluded, such as post-translational modification (*BraA10g029710*, encoding a protein phosphatase 2C family protein) and signal transduction (*BraA10g029790* and *BraA10g029880*, both encoding receptor-like kinase family proteins). Future work should focus on narrowing down the candidate interval and functionally validating the candidate genes through approaches such as genetic complementation, overexpression, or gene editing. Our study thus lays a foundation for future fine mapping of the candidate gene within the identified interval on chromosome A10, and provides new insights into the molecular basis of the purple coloration in leaf veins, petioles, and flowering stalks in *B. rapa*.

## 4. Materials and Methods

### 4.1. Plant Materials and Growth Conditions

The *B. rapa* inbred line 7A01 and cultivar ‘Jinqiu 66’, as well as the F_1_, F_2_, and BC_1_ segregation population, were planted at the experimental farm of Northwest A&F University (Yangling, Shaanxi, China) in late September of 2024 and 2025. At the five-leaf stage and the bolting stage, the purple coloration trait was recorded, and DNA was extracted for gene mapping.

### 4.2. Photometric Determination of Anthocyanin Contents

The measurement of anthocyanin contents was carried out as previously reported, with minor modifications [56]. For each genotype, three biological replicates were sampled from the third true leaf at the five-leaf stage and from the fifth true leaf at the bolting stage. The sample to be analyzed was thoroughly ground in liquid nitrogen. Then, 0.3 mL of the extraction solution (methanol solution containing 1% hydrochloric acid) was added, and the mixture was mixed evenly and incubated in the dark at 4 °C overnight. Subsequently, 0.2 mL of distilled water and 0.5 mL of chloroform were added, and the sample was thoroughly vortexed for 40 s and centrifuged at 10,000 rpm for 4 min at room temperature. The absorption at wavelengths of 530 nm and 657 nm in the aqueous phase was measured using a spectrophotometer (V-1200, Mapada, Shanghai, China). The calculation formula for the relative content of anthocyanins is as follows: relative anthocyanin content = (A_530_ − 0.25 × A_657_)/Sample fresh weight (g).

### 4.3. Chlorophyll Measurement

Chlorophyll content was determined using a portable chlorophyll meter (SPAD-502, Konica Minolta, Osaka, Japan). The measurement is based on the differential transmittance of light at 650 nm (red) and 940 nm (infrared), from which the SPAD value is calculated as a relative indicator of leaf chlorophyll content. Measurements were conducted on clear sunny days between 9:00 and 11:00 a.m. For each genotype, six biological replicates were sampled from the third true leaf at the five-leaf stage and from the fifth true leaf at the bolting stage. SPAD readings were recorded from the middle portion of each leaf blade, avoiding the main vein.

### 4.4. Measurement of Net Photosynthesis Rate and Intercellular CO_2_ Concentration

Photosynthetic parameters were measured using a portable photosynthesis system (LI-6400/XT, LI-COR, Lincoln, NE, USA). Measurements were conducted on clear sunny days between 9:00 and 11:00 a.m. For each genotype, six biological replicates were sampled from the third true leaf at the five-leaf stage and from the fifth true leaf at the bolting stage. During the measurements, the leaf chamber CO_2_ concentration was maintained at 400 μmol·mol^−1^ using a high-pressure CO_2_ cylinder, the system pressure was set to 101 kPa, and the flow rate was controlled at 500 μmol·s^−1^. The net photosynthetic rate (*Pn*) and intercellular CO_2_ concentration (*Ci*) were recorded for each leaf. All measurements were performed on the middle portion of the leaf blade, avoiding the main vein.

### 4.5. DNA Extraction and SNP Genotyping

Single-nucleotide polymorphism (SNP) genotyping was performed with a *Brassica* 50 K Illumina Infinium™ SNP array (Greenfafa, Wuhan, China). Total genomic DNA was extracted from fresh leaves of the two parents (15 individual plants each for 7A01 and ‘Jinqiu 66’) and the F_2_ population (30 individual plants with purple flowering stalk and 30 with green flowering stalk) with a plant genomic DNA extraction kit (Tiangen Biotech, Beijing, China) following the manufacturer’s protocol. Bulk DNA samples were constructed with an equivalent amount of DNA from 10 individuals of each parent and 30 individuals from the F_2_ population. Consequently, four bulk DNA samples, representing two parents, F_2_ plants with purple flowering stalks, and F_2_ plants with green flowering stalks, were used for SNP genotyping with a *Brassica* 50 K Illumina Infinium™ SNP array as previously described [57]. Meanwhile, the gene sequences were subjected to BLAST alignment (https://blast.ncbi.nlm.nih.gov/Blast.cgi, accessed on 20 June 2025), and the resulting data were analyzed for synteny using TBtools software (Version 2.301).

### 4.6. Molecular Marker Development, Linkage Analysis, and Locus Mapping

Based on the genome assemblies of the *Brassica* database (BRAD: http://brassicadb.cn/#/, accessed on 25 June 2025) [58,59], SSR and IP molecular markers were developed. The SSR primers were designed using the SSRHunter software V1.3.0 (Nanjing Agricultural University, Nanjing, China). Meanwhile, the IP primers were designed using Primer Premier 5.0 software (PREMIER Biosoft, San Francisco, CA, USA). The resulting primer sequences were synthesized by Sangon Biotech (Shanghai, China). The primers are listed in Supplementary Table S4.

### 4.7. RNA-Seq and Data Analysis

For RNA-seq, shoot apices at the five-leaf stage and flowering stalks at the bolting stage were harvested, each with three biological replicates, with each biological replicate composed of five randomly selected individual plants. For shoot apices, RNA library construction, sequencing, and data analysis were performed by LC-Bio Technologies (Hangzhou, China) (https://www.omicstudio.cn/home?slide=3, accessed on 10 December 2025). The quality of transcriptome data was checked, and principal components analysis and Pearson correlation analysis were performed to evaluate sample clustering and overall data reliability (Supplementary Figure S2 and Table S5). The reference genome was *B. rapa* Chiifu V2.5 (http://brassicadb.cn/#/Download/, accessed on 10 December 2025). DEGs were identified using a threshold of |log_2_(fold change)| ≥ 1 and false discovery rate (FDR) < 0.05. Identified DEGs were submitted to the Bioinformatics Online Tool (https://www.bioinformatics.com.cn/, accessed on 11 December 2025) for Gene Ontology (GO) enrichment analysis, and significantly enriched GO terms were retrieved.

For flowering stalks, RNA library construction, sequencing, and data analysis were carried out by GENE DENOVO (Guangzhou, China) (https://www.omicsmart.com/#/platform?id=4, accessed on 12 March 2024). Similarly, the quality of transcriptome data was checked, and principal components analysis and Pearson correlation analysis were performed to evaluate sample clustering and overall data reliability (Supplementary Figure S3 and Table S6). The reference genome was *B. rapa* Chiifu V3.0 (http://brassicadb.cn/#/Download/, accessed on 12 March 2024). DEGs were filtered with the same criteria of |log_2_(fold change)| ≥ 1 and false discovery rate (FDR) < 0.05. Homologous *A. thaliana* genes corresponding to each *B. rapa* DEG were obtained using local BLASTP searches. GO enrichment analysis was subsequently performed using the DAVID database (https://davidbioinformatics.nih.gov/, accessed on 14 March 2024).

### 4.8. RT-qPCR Analysis

Total RNA was extracted from shoot apices at the five-leaf stage and flowering stalks at the bolting stage using the MiniBEST Plant RNA Extraction Kit (TaKaRa Bio, Dalian, China). RNA integrity was assessed by agarose gel electrophoresis, and RNA concentration and purity were determined using a NanoDrop One spectrophotometer (Thermo Fisher Scientific, Waltham, MA, USA). First-strand cDNA was synthesized with the PrimeScript RT Mix (TaKaRa Bio, Dalian, China). RT-qPCR was performed using the TB Green Premix Ex Taq II (TaKaRa Bio, Dalian, China). *BraGAPDH* was used as the internal reference gene. Each reaction was performed with three biological replicates and three technical replicates. Primers used for RT-qPCR are listed in Supplementary Table S4.

### 4.9. Statistical Analysis

Student’s *t*-tests and ANOVA were performed using GraphPad Prism 8.0.

## Figures and Tables

**Table 1:** Genetic analysis of the purple coloration trait.

Years	Population	Total	Green Coloration	Purple Coloration	Expectations	X^2^	*p*
2024	Jinqiu 66 (P_1_)	100	100	0	—	—	—
	7A01 (P_2_)	100	0	100	—	—	—
	F_1_	100	0	100	—	—	—
	Reciprocal (F_1_)	100	0	100	—	—	—
	F_2_	270	67	203	1:3	0.005	0.943
2025	Jinqiu 66 (P_1_)	100	100	0	—	—	—
	7A01 (P_2_)	100	0	100	—	—	—
	F_1_	100	0	100	—	—	—
	Reciprocal F_1_	100	0	100	—	—	—
	BC_1_	615	308	307	1:1	0.002	0.968
	F_2_	469	122	347	1:3	0.257	0.613

## Data Availability

The original contributions presented in this study are included in the article/Supplementary Materials. Further inquiries can be directed to the corresponding authors.

## Supplementary Materials

The following supporting information can be downloaded at: https://www.mdpi.com/article/10.3390/plants15142192/s1: Figure S1. The stalk color characteristics of Jinqiu 66 (green stalk), 7A01 (purple stalk), and their F_1_ hybrid offspring. Scale bars = 2 cm. Figure S2. Principal components analysis (A) and Pearson correlation analysis (B) of transcriptome data of shoot apices from Jinqiu 66 and 7A01 at the five-leaf stage. Figure S3. Principal components analysis (A) and Pearson correlation analysis (B) of transcriptome data of flowering stalks from Jinqiu 66 and 7A01 at the bolting stage. Table S1. Polymorphism SNP_S_ between the green color samples and purple color samples obtained by *Brassica* 50K Illumina Infinium™. Table S2. Collinearity analysis results. Table S3. List of 46 genes on chromosome A10 within the localized interval. Table S4. Primers used in this study. Table S5. Summary of transcriptome data of shoot apices from Jinqiu 66 and 7A01 at the five-leaf stage. Table S6. Summary of transcriptome data of flowering stalks from Jinqiu 66 and 7A01 at the bolting stage.
