# Supplementary material for: Genetic and Transcriptomic Analyses of the Purple Coloration Trait in the Inbred Line 7A01 of Brassica rapa
Source: Plants (Basel). 2026 Jul 17;15(14):2192. doi: 10.3390/plants15142192 (PMC13417351; doi:10.3390/plants15142192)
Supplement: Supplementary file 1 [file plants-15-02192-s001.zip › plants-4321852-supplementary/plants-4321852 Supplementary files/plants-4321852 Supplementary Figures.pdf]

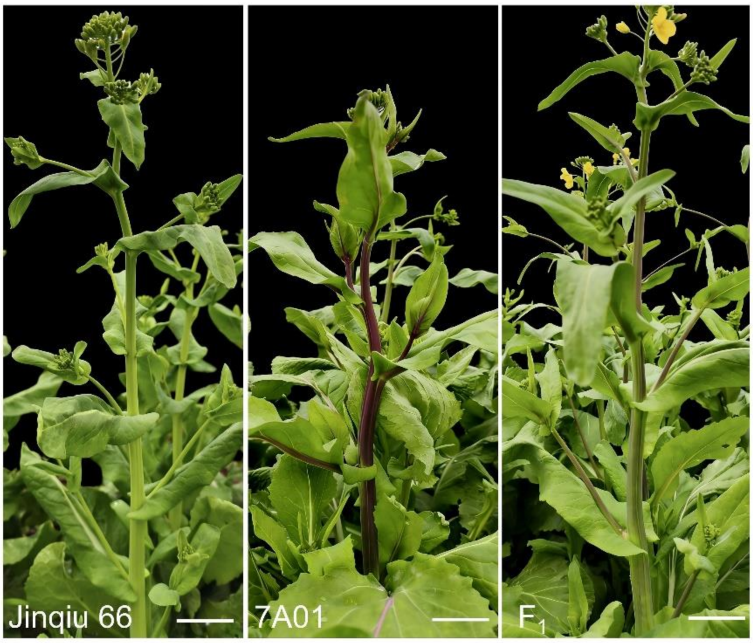

Supplementary Figure S1. The stalk color characteristics of Jinqiu 66 (green stalk), 7A01 (purple stalk), and their F_1 hybrid offspring. Scale bars = 2 cm.

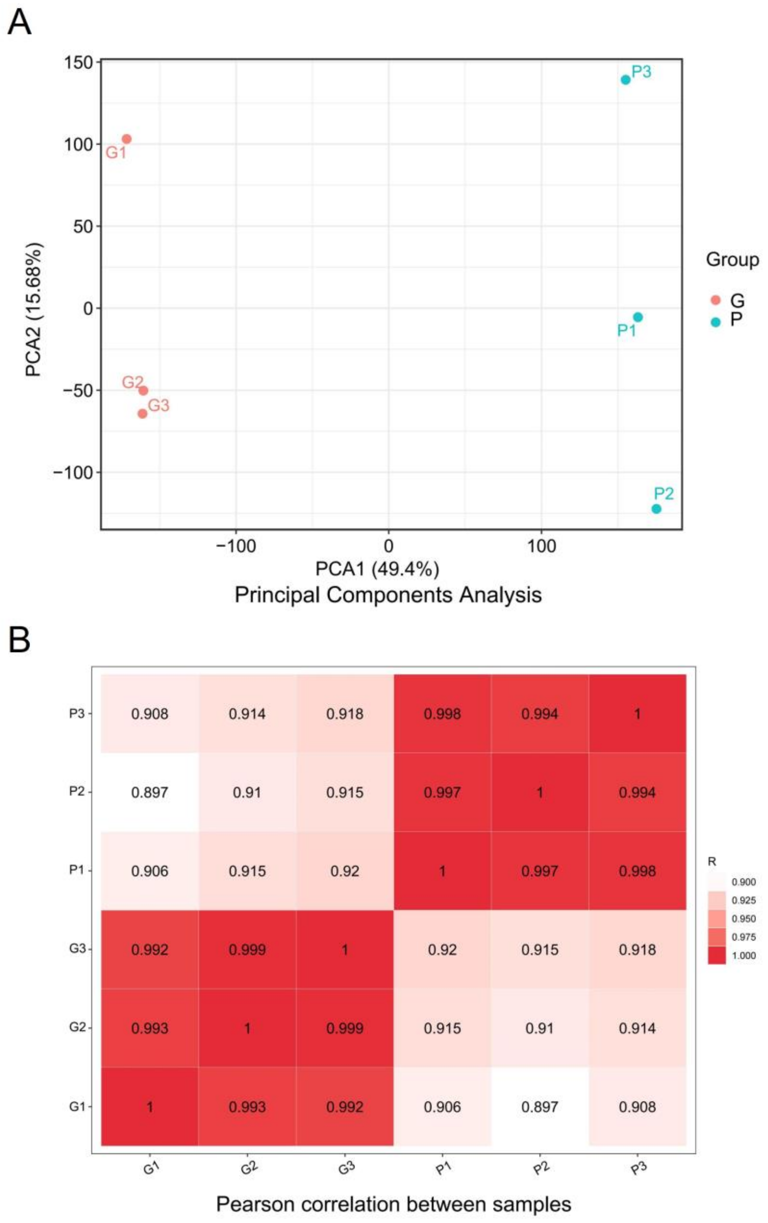

Supplementary Figure S2. Principal components analysis (A) and Pearson correlation analysis (B) of transcriptome data of shoot apices from Jinqiu 66 and 7A01 at the five-leaf stage. G1, G2, and G3 represent biological replicates of Jinqiu 66. P1, P2, and P3 represent biological replicates of 7A01.

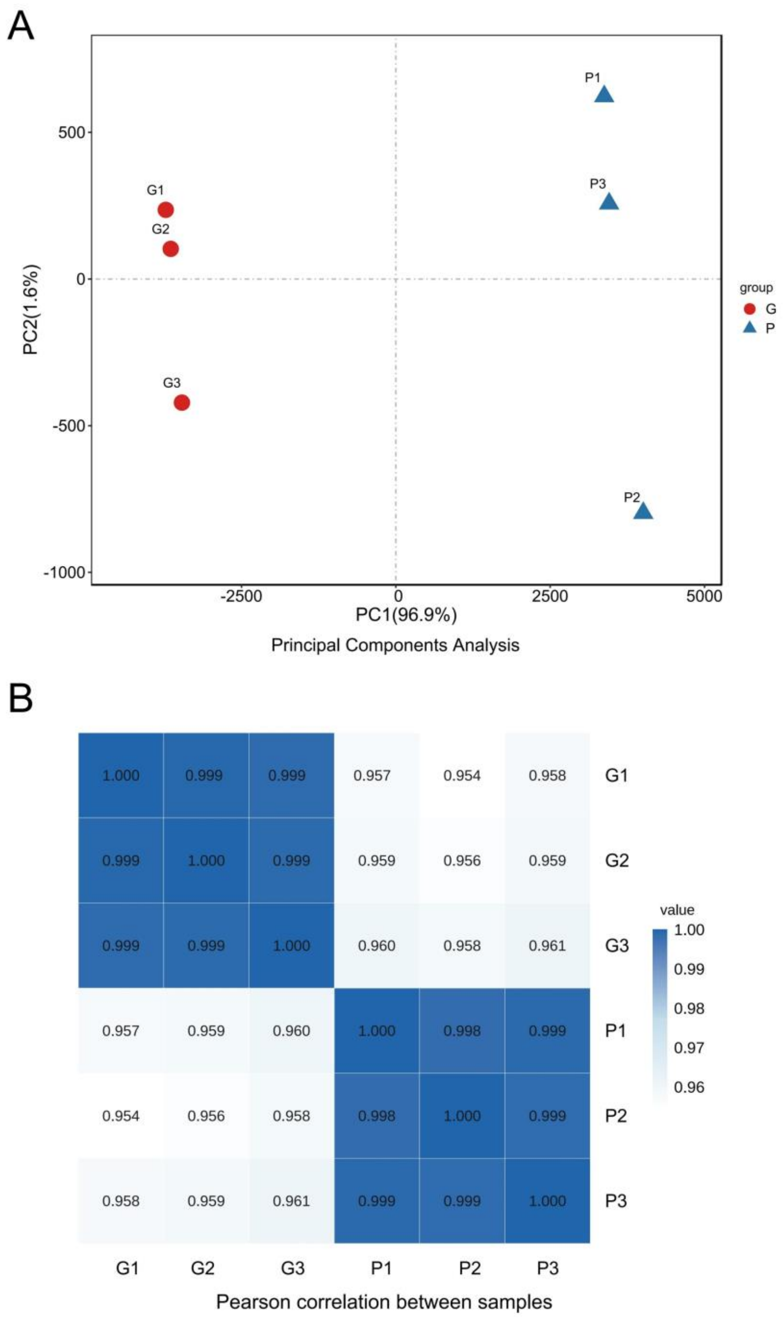

Supplementary Figure S3. Principal components analysis (A) and Pearson correlation analysis (B) of transcriptome data of flowering stalks from Jinqiu 66 and 7A01 at the bolting stage. G1, G2, and G3 represent biological replicates of Jinqiu 66. P1, P2, and P3 represent biological replicates of 7A01.
