# Supplementary material for: Genetic and Transcriptomic Analyses of the Purple Coloration Trait in the Inbred Line 7A01 of Brassica rapa
Source: Plants (Basel). 2026 Jul 17;15(14):2192. doi: 10.3390/plants15142192 (PMC13417351; doi:10.3390/plants15142192)
Supplement: Supplementary file 1 [file plants-15-02192-s001.zip › plants-4321852-supplementary/plants-4321852 Supplementary files/plants-4321852 Supplementary Tables.pdf]

Supplementary Table S1. Polymorphism SNPs between the green color samples and purple color samples obtained by *Brassica* 50K Illumina Infinium™

SNP list	Chromosome	Location	Green color	Purple color
BBn-A01-p1000115	chrA01	15651739	A	B
BBn-scaff_17369_1-p83617	chrA10	4431137	A	B
Bseq-new-rs25345	chrA10	4453506	A	B
Bseq-new-rs48035	chrA10	4459519	A	B

Supplementary Table S2. Collinearity analysis results

Genome (*B. rapa*)	Genes number (*B. rapa*)	Gene location
A01	8	
A02	7	
A03	10	
A04	2	
A06	1	
A08	2	
A09	1	
A10	192	28153510-29262541

Supplemental Table S3. List of 46 genes on chromosome A10 within the localized interval

Gene ID	Arabidopsis ID	Description
BraA10g028970	*AT5G05900*	Glycosyltransferase involved in the biosynthesis of the guanine hexoside derivatives.
BraA10g029010	*AT1G11610*	Putative cytochrome P450.
BraA10g029040	*AT5G05940*	Encodes a member of KPP-like gene family.
BraA10g029050	*AT5G05950*	Maternal effect embryo arrest 60.
BraA10g029060	*AT5G05960*	Involved in adventitious root organogenesis.
BraA10g029070	*AT5G05965*	Cell wall RBR3-like protein.
BraA10g029090	*AT5G05980*	Encodes one of the three folylpolyglutamate synthetase isoforms.
BraA10g029100	*AT5G05990*	Mitochondrial glycoprotein family protein.
BraA10g029080	*AT5G05970*	A WD40 repeat protein related to the animal NEDD1/GCP-WD protein.
BraA10g029110	*AT5G06050*	Putative methyltransferase family protein.
BraA10g029120	*AT5G06060*	NAD(P)-binding Rossmann-fold superfamily protein.
BraA10g029470	*AT5G06470*	Glutaredoxin family protein.
BraA10g029480	*AT5G06480*	Immunoglobulin E-set superfamily protein.
BraA10g029490	*AT5G06510*	Enrichment of functional NFYA10.1 variant is triggered by phosphorylated SRRML1.
BraA10g029990	*AT5G07550*	Member of Oleosin-like protein family.
BraA10g030030	*AT5G07500*	Encodes an embryo-specific zinc finger transcription factor required for heart-stage embryo formation.
BraA10g030040	*AT5G07480*	KAR-UP oxidoreductase 1.
BraA10g029500	*AT5G52820*	Encodes a NOTCHLESS homolog.
BraA10g029830<br>*BraA10g029840*<br>*BraA10g029850*<br>*BraA10g029860*	*AT5G06860*	Encodes a polygalacturonase-inhibiting protein involved in defense response.
BraA10g029870	*AT5G06930*	Nucleolar-like protein.
BraA10g029880.2<br>*BraA10g029880.4*	*AT5G06940*	Leucine-rich repeat receptor-like protein kinase family protein.
BraA10g030000	*AT5G07540*	Encodes a glycine-rich protein that is expressed only in flowers during a specific developmental stage.

BraA10g030010	*AT5G07530*	Encodes a glycine-rich protein that has oleosin domain and is expressed specifically during flower stages 10 to 12.
BraA10g030050	*AT5G07470*	Ubiquitous enzyme that repairs oxidatively damaged proteins.
BraA10g030060	*AT5G07460*	Ubiquitous enzyme that repairs oxidatively damaged proteins.
BraA10g029560	*AT5G06590*	Hypothetical protein.
BraA10g029600	*AT5G03495*	RNA-binding (RRM/RBD/RNP motifs) family protein.
BraA10g029630	*AT5G06680*	Encodes protein similar to yeast SCP98.
BraA10g029670	*AT5G06720*	Encodes a peroxidase with diverse roles in the wound response, flower development, and syncytium formation.
BraA10g029680	*AT5G06730*	Peroxidase superfamily protein.
BraA10g029690	*AT1G09700*	Encodes a nuclear dsRNA binding protein. Involved in mRNA cleavage.
BraA10g029700	*AT5G06740*	Concanavalin A-like lectin protein kinase family protein.
BraA10g029710	*AT5G06750*	Protein phosphatase 2C family protein.
BraA10g029820	*AT5G06850*	Encodes an endoplasmic reticulum protein.
BraA10g029720	*AT5G06760*	Encodes LEA4-5, a member of the Late Embryogenesis Abundant (LEA) proteins.
BraA10g029730 *BraA10g029740*	*AT5G06790*	Encodes cotton fiber protein.
BraA10g029760	*AT4G34710*	Encodes an arginine decarboxylase (ADC), a rate-limiting enzyme.
BraA10g029770	*AT5G06800*	Member of the PHR1-LIKE (PHL) transcription factor family.
BraA10g029780	*AT5G06810*	Transcription termination factor family protein.
BraA10g029790	*AT5G06820*	STRUBBELIG-receptor family 2.
BraA10g029810	*AT5G06839*	As part of miR319-TCPs-TGA9/TGA10/ROXY2 regulatory module controls cell fate specification in early anther development.

Supplementary Table S4. Primers used in this study

Primer name	Primer sequence (5'→3')	Annotation
IP50-F	AGCTTGGCCGGGAGGTAATGAGG	IP marker
IP50-R	CTCCAATCAATCACAAGCTTGCC	
IP60-F	AGGAAGAAGAAGCCATGATGGAC	IP marker
IP60-R	GCAAGATCATTGTCTGGATCAAG	
IP80-F	ATCCATCTTCCGGACGATCTCAC	IP marker
IP80-R	GCGGTGACAATAATAACACACTC	
SSR46-F	GGAACTGAAACTAAGGGACAAGGCC	SSR marker
SSR46-R	GACGAAGAATCAAAACGGCCAC	
SSR91-F	TCTTTCGTCACCATCCAT	SSR marker
SSR91-R	AGAAGCAGACAGACCGAG	
SSR197-F	AAGAGTTTCCACCCATCC	SSR marker
SSR197-R	GAGTCGGTGATTCTTTCC	
SSR205-F	AAGTTATTGGAATTGGTT	SSR marker
SSR205-R	TGAAACTCGTCCCATAAG	
SSR241-F	AGATGAACAGCGGAGCAGAG	SSR marker
SSR241-R	CTGGCTGAGGAACAGAAACA	
BraA06.UF3GT-1-F	ACCTCTACCCAAATCTCATA	RT-qPCR
BraA06.UF3GT-1-R	TGTCTCCTCCAGACGAAG	
BraA01.ANS-2-F	AAGGCGGCTATGGACTGG	RT-qPCR
BraA01.ANS-2-R	CTGAGGGCATTTCGGGTA	
BraA04.PAL3-1-F	AAGTTTGTGAGGGAAGAGC	RT-qPCR
BraA04.PAL3-1-R	AACAGATGGGAATGGGAG	
BraA05.PAL3-F	ACTCACCGACTCAAACATCA	RT-qPCR
BraA05.PAL3-R	CTCCGAGAACTGAGCAAA	
BraA07.CHI-F	GTCCAACACCGTTACCCT	RT-qPCR
BraA07.CHI-R	ACCGATGACCGTGAAGAT	
BraA09.CHS-F	ACTACTACTTCCGCATCACC	RT-qPCR
BraA09.CHS-R	GGGACTTCAACCACCACTA	
BraA04.C4H-F	GCGAACACTGGCGTAAGA	RT-qPCR
BraA04.C4H-R	GCAAGCGTTTCCTCAATA	
BraA10.HY5-F	TCCCTACCGTCAAGCAGC	RT-qPCR
BraA10.HY5-R	CGTTAGAACCACCGCCTC	
BraA06.PAP3-F	ACCCGTGGATGAAGTTGT	RT-qPCR

BraA06.PAP3-R	AAGGGATTGATGATGAGGAT	
BraA04.PAL3-2-F	ACTCCTCCGCCGCTAATC	RT-qPCR
BraA04.PAL3-2-R	TGCACAGACCTGCCCAAC	
BraA05.4CL1-F	ACTCACGAGCAACGGTAT	RT-qPCR
BraA05.4CL1-R	CATCAATCAACAAAGGGT	
BraA05.4CL5-F	ACCCACCCACCGCATCTA	RT-qPCR
BraA05.4CL5-R	ACCGCAATCCTCACCTTCTC	
BraA09.DFR-F	TACACCTCTGGGTTCTCA	RT-qPCR
BraA09.DFR-R	TTTACCTTCAATGCTCCT	
BraA01.ANS-1-F	TACTATTGAACCGCCACA	RT-qPCR
BraA01.ANS-1-R	CGAGTTACCATCAGCCAC	
BraA03.ANS-F	GTGGTTTCCCTTTACTGC	RT-qPCR
BraA03.ANS-R	TGAGGTTGACTATTGTGGG	
BraA06.UF3GT-2-F	GATGAGCCGTCAAGAGGT	RT-qPCR
BraA06.UF3GT-2-R	AGGAGGAGATAAGGGATG	
BraA09.5MAT-F	GATGAGCCGTCAAGAGGT	RT-qPCR
BraA09.5MAT-R	AGGAGGAGATAAGGGATG	
BraA02.TT19-F	TGAAATCGGTGGAGGTAA	RT-qPCR
BraA02.TT19-R	CCAGTGTCAAATGGGTAG	
BraGAPDH-F	CCACTTGCCAAGGTTATCAACGAC	Internal
BraGAPDH-R	CAACTGAAACATCAACGGTGGG	control

Supplemental Table S5. Summary of transcriptome data of shoot apices from Jinqiu 66 and 7A01 at the five-leaf stage

Sample	Raw reads	Clean reads	Mapping reads	Mapping rate(%)	GC(%)
G1	41561512	40334536	35323026	87.58	47
G2	42479518	41324806	36192328	87.58	47
G3	43298190	42005440	36742299	87.47	47
P1	44676622	43299598	37911604	87.56	47
P2	38685126	37433484	32727379	87.43	47
P3	39867846	38651600	33864792	87.62	47

Supplemental Table S6. Summary of transcriptome data of flowering stalks from Jinqiu 66 and 7A01 at the bolting stage

Sample	Raw reads	Clean reads	Mapping reads	Mapping rate(%)	GC(%)
G1	53063116	52849534	46316165	87.64	47.82
G2	51830506	51597842	45256510	87.71	47.71
G3	40657918	40503748	35419357	87.45	47.74
P1	51452096	51182114	44402621	86.76	47.55
P2	47284146	47025776	40904770	86.99	47.61
P3	47877804	47646430	41618394	87.35	47.59
